# Malaria in Kakuma refugee camp, Turkana, Kenya: facilitation of *Anopheles arabiensis *vector populations by installed water distribution and catchment systems

**DOI:** 10.1186/1475-2875-10-149

**Published:** 2011-06-04

**Authors:** M Nabie Bayoh, Willis Akhwale, Maurice Ombok, David Sang, Sammy C Engoki, Dan Koros, Edward D Walker, Holly A Williams, Heather Burke, Gregory L Armstrong, Martin S Cetron, Michelle Weinberg, Robert Breiman, Mary J Hamel

**Affiliations:** 1 Centre for Global Health Research, Kenya Medical Research Institute/Centres for Disease Control and Prevention, P.O. Box 1578, Kisumu, Kenya; 2 Division of Malaria Control, Ministry of Health, Nairobi, Kenya; 3 Kenya Methodist University, Meru, Kenya; 4 International Rescue Committee, Kakuma Refugee Camp, Kenya; 5 Department of Microbiology and Molecular Genetics, Michigan State University, East Lansing, Michigan 48824, USA; 6 International Emergency and Refugee Health Branch, Centres for Disease Control and Prevention, 1600 Clifton Rd, Atlanta, Georgia 30333, USA; 7 Centres for Disease Control and Prevention, International Emerging Infections Programme, Mbagathi Way, Nairobi, Kenya; 8 Centers for Disease Control and Prevention, Malaria Branch, 1600 Clifton Road, Mailstop F-22, Atlanta GA 30301, USA

## Abstract

**Background:**

Malaria is a major health concern for displaced persons occupying refugee camps in sub-Saharan Africa, yet there is little information on the incidence of infection and nature of transmission in these settings. Kakuma Refugee Camp, located in a dry area of north-western Kenya, has hosted ca. 60,000 to 90,000 refugees since 1992, primarily from Sudan and Somalia. The purpose of this study was to investigate malaria prevalence and attack rate and sources of *Anopheles *vectors in Kakuma refugee camp, in 2005-2006, after a malaria epidemic was observed by staff at camp clinics.

**Methods:**

Malaria prevalence and attack rate was estimated from cases of fever presenting to camp clinics and the hospital in August 2005, using rapid diagnostic tests and microscopy of blood smears. Larval habitats of vectors were sampled and mapped. Houses were sampled for adult vectors using the pyrethrum knockdown spray method, and mapped. Vectors were identified to species level and their infection with *Plasmodium falciparum *determined.

**Results:**

Prevalence of febrile illness with *P. falciparum *was highest among the 5 to 17 year olds (62.4%) while malaria attack rate was highest among the two to 4 year olds (5.2/1,000/day). Infected individuals were spatially concentrated in three of the 11 residential zones of the camp. The indoor densities of *Anopheles arabiensis*, the sole malaria vector, were similar during the wet and dry seasons, but were distributed in an aggregated fashion and predominantly in the same zones where malaria attack rates were high. Larval habitats and larval populations were also concentrated in these zones. Larval habitats were man-made pits of water associated with tap-stands installed as the water delivery system to residents with year round availability in the camp. Three percent of *A. arabiensis *adult females were infected with *P. falciparum *sporozoites in the rainy season.

**Conclusions:**

Malaria in Kakuma refugee camp was due mainly to infection with *P. falciparum *and showed a hyperendemic age-prevalence profile, in an area with otherwise low risk of malaria given prevailing climate. Transmission was sustained by *A. arabiensis*, whose populations were facilitated by installation of man-made water distribution and catchment systems.

## Background

A strong association exists between human uses of water, production of adult malaria vectors from aquatic environments containing the larval stages, and subsequent malaria transmission [1]. The societal requirements for irrigation, dams for electrical energy generation, for watering animals, and for other domestic uses can create a paradoxical intensification of pathogen transmission through vector habitat formation and subsequent production of adult stages of vectors [2-5]. This relationship becomes particularly important in arid environments where water often must be channelled and retained for domestic and agricultural uses and to avoid water loss. For example, a program of micro-dam construction in Ethiopia resulted in increased malaria prevalence [6]. Hunter *et al *[7] called for intersectoral negotiation when a conflict between water resource development and parasitic diseases such as malaria emerges, in order to predict, interdict, and alleviate the increased burden of disease that ensues as development proceeds.

Malaria is a major health concern for refugees living in camps in sub-Saharan Africa [8,9]. Despite the long term presence of refugee settlements, there is little information on malaria in the camps, patterns of transmission, or effectiveness of malaria control [9]. Kakuma refugee camp was established in 1992 in an arid region of north-western Kenya near the border with Sudan, primarily to accommodate Sudanese refugees fleeing a civil war [10]. At the time of this study in 2005-2006, the camp hosted approximately 90,000 refugees, comprised of 78% Sudanese, 14% Somali, 3% Ethiopians, and the remaining 4% from seven other sub-Saharan African countries [11]. Kakuma refugee camp is administered by the United Nations High Commissioner for Refugees (UNHCR), assisted by several nongovernmental organizations (NGOs) referred to as "implementing partners". The main implementing partners in the camp included the International Rescue Committee (IRC), which was responsible for health and sanitation in the camp, the World Food Programme (WFP), Jesuit Refugee Services (JRS), German Cooperative Agency (GTZ), FilmAid International, National Council of Churches of Kenya (NCCK), and Lutheran World Federation (LWF).

As lead health implementing partner, IRC was responsible for malaria control interventions, including vector control and case management. The vector control activities in the camp included indoor residual spraying, larval control using a synthetic oil, distribution of insecticide-treated nets to pregnant women at first antenatal clinic visit and children under five years of age at maternal and child health visits, and intermittent insecticidal fogging of the premises of schools, hospitals and other institutions. Kakuma refugee camp maintains four free medical clinics, open Monday through Saturday, and a 90-bed, in-patient referral hospital with surgical capacity. The LWF had responsibility for the camp water supply including operating and maintaining approximately 255 tap-stands where residents collected water. In the early 2000s, the GTZ introduced a kitchen garden concept into the camp, whereby residents were encouraged to make small vegetable gardens within the compounds of the various service providers in the camp and around residential units. To facilitate irrigation of the kitchen gardens, GTZ staff constructed pits in the vicinity of each tap-stand where run-off water from the tap-stands accumulated. Each tap-stand in the camp had between one to three pits, referred to here as tap-stand pits, which were either cemented or not cemented (i.e., left with a natural soil lining). Small drainage channels - cemented or soil-lined - were created to capture run-off water originating around the tap-stands, and to direct the water into the tap-stand pits.

In June-August, 2005, the Kakuma refugee camp and its environs experienced a malaria epidemic associated with the annual rainy season [12]. In early July, the number of patients presenting to the clinics with clinically-diagnosed malaria increased substantially, with approximately 11,000 cases seen, corresponding to a 12.2% attack rate (D. Koros, personal observation; Kakuma clinic staff, personal communication). The case fatality rate was not determined but 13 deaths associated with malaria clinical diagnosis were observed by a staff physician [12]. *Plasmodium falciparum *malaria prevalence was investigated in febrile patients presenting to clinics in the camp in August 2005, immediately after the epidemic, and two months after the end of the rainy season. The malaria survey was followed by investigations on the sources of the *Anopheles *vectors in the camp with surveys conducted in February 2006, during the dry season, and in June 2006, during the rainy season. Entomologic surveys at these two time points were designed to understand the temporal dynamics of the local vector populations and the habitats that produce them to investigate transmission patterns in the area.

## Methods

### Study site

**Figure 1:**
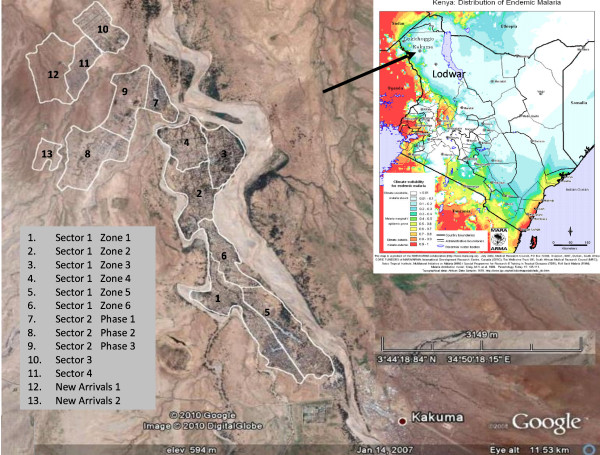
**Google earth image and overlaid map of Kakuma refugee camp showing the divisions of the camp**. Inset: MARA ARMA map of Kenya showing distribution on endemic malaria in the country, and the location of Kakuma.

Kakuma refugee camp is situated near Kakuma, Turkana District, in the semi-arid north-west region of Kenya (Figure 1). The camp lies between latitude 3°42'N and 3°46'N and longitude 34°51'E and 34°49'E. Kakuma is hot and arid, with prolonged dry seasons and low rainfall (~200 mm per year on average). The climatic conditions are inhospitable for malaria vectors for most of the year; malaria transmission in the area occurs sporadically in phase with the arrival of the annual rains. The Kenyan population, living adjacent to the camp, consists of the nomadic and pastoralist Turkana. The camp is near a dry river bed that is prone to flash flooding after heavy rains in Uganda, even when Kakuma itself receives no rain. The local Turkana people often excavate wells along the river bed to water their livestock during dry season or frequently migrate across expansive geographical areas to find water and vegetation. Malaria distribution maps based upon climate patterns predict that this area has marginal to low malaria transmission with epidemic potential after excessive rainfall (Figure 1) [13,14].

Most residents of Kakuma refugee camp were housed in mud brick dwellings while some lived in temporary shelters, including tents. The camp was divided administratively into four large sectors, called Kakuma Sectors 1- 4 (Figure 1). Sector 1 was divided into six Zones (referred to as Zones 1-6), and Kakuma Sector 2 was divided into three Phases. Sectors, Zones or Phases were further sub-divided into blocks, and blocks into households. The population per block ranged from 500 to 4,000 people.

A total of three surveys were carried out; the malaria prevalence survey in August 2005, which is a dry period in the camp, the dry season entomological survey in February 2006 and the wet season entomological survey in June 2006.

### Malaria prevalence survey

To estimate prevalence of clinical malaria (defined as laboratory confirmed *Plasmodium *infection plus axillary temperature ≥ 37.3°C) from patients presenting to the camp clinics, a survey was conducted over a 5-day period, in August 2005. All patients over six months of age presenting to any of the camp's clinics with an axillary temperature of at least 37.3°C were tested for malaria infection after obtaining a verbal consent. Demographic information, travel history and recent anti-malarial use were recorded for each febrile patient, and a blood sample taken for preparation of a blood smear and malaria rapid diagnostic test performed (RDT; ParaCheck^®^, Orchid Biomedical Systems, Vema Goa, India). Haemoglobin concentrations were measured using HemoCue^R ^B analyzers (Ängelholm, Sweden). Patients with a positive RDT were treated by clinic staff. Blood smears were read at a later date by an experienced microscopist blinded to the RDT results. Parasite densities were estimated with the standard method by counting the number of trophozoites per 300 white blood cells in the thick smear and assuming a white blood cell count of 8,000 per microlitre of blood.

### Larval habitat survey

For mosquito sampling, Kakuma refugee camp was divided into 13 clusters corresponding to the various land units as shown in Figure 1. Eleven out of 13 clusters during dry season survey and 10 out of the 13 clusters during the wet season survey. These selected clusters represented stable residences for the refugee population that excluded make shift tents or houses where new arrivals to the camp temporarily stayed during registration or before they were allocated a house. During the dry season survey, which was the first entomological survey, a field investigation revealed the presence of water sources configured as tap-stands (pipes with hand valves), often with associated water-filled, ground pits. Many contained *Anopheles *larvae. Therefore, all tap-stand associated and non-tap-stand associated water sources in each selected cluster were located and mapped by ground reconnaissance. Each water source was geo-referenced using hand-held differential global positioning system equipment (GPS, Trimble Navigation Ltd, California, and USA) which was also used for recording data at each habitat. The habitats were sampled for immature stages of malaria vectors by taking one standard collection of water (450 ml) with a mosquito dipper per linear meter of habitat edge, larval counts were totaled, and the average number per dip per habitat was calculated.

### Adult mosquito survey

Adult vector surveillance was performed using pyrethrum spray collections (PSC) with 0.025% pyrethrum and 0.1% piperonyl butoxide synergist in paraffin (kerosene) as described elsewhere [15]. It was done with randomly selected clusters of houses as follows. Seven index houses in the dry season survey and 10 in the wet season survey were purposively selected, so that most of the residential areas of the camp were covered. Then, the 30 nearest houses to each index house were selected for PSC. Sampled mosquitoes were placed in labelled petri dishes and morphologically identified to species level. All *Anopheles *adults were kept in individual tubes and preserved using drierite for species identification by a modified polymerase chain reaction method [16]. Infection with *P. falciparum *sporozoites was determined on individual mosquitoes by the method of Wirtz *et al *[17].

### Rainfall

Monthly rainfall data were obtained from the Lodwar meteorological station from 2000 to 2007. Data were averaged and the 95% confidence interval around each monthly mean calculated and compared graphically with the monthly rainfall in 2005.

### Data analysis

Geo-referenced data were downloaded with differential correction into a GPS database (GPS Pathfinder Office 2.8, Trimble Navigation Ltd, California, USA) alongside the corresponding vector data. The database was exported as dbase files to SAS version 9.1 (SAS Institute 2000-2004) for statistical analysis and to Arc GIS 9.2 (ESRI, Redlands, California) for spatial visualization. Chi-square tests were used to determine the differences in *P. falciparum *positive rates between categories (age and location) of patients identified during the malaria surveillance and among the proportions of habitats and houses positive for *Anopheles *and *Culex *larvae and adults between the dry season and the wet season entomological surveys. Poisson regression was used to compare *Anopheles *larva density and *Anopheles *and *Culex *adult densities between the dry and wet seasons. Generalized estimating equations (GEE) with an exchangeable working correlation matrix was used to control for correlation at the habitat and house levels, respectively, in the Poisson regression models (PROC GENMOD in SAS). Poisson regression was also used to model adult mosquito density with the number of breeding sites. GEE was used to control for correlation at the site level. Maps were generated to depict the distribution pattern of both *Anopheles *larva and adult during the two seasons. To estimate the attack rate of clinical malaria among camp residents, we included all patients presenting to the camp clinics over a 3 day period when all clinics were open to the public. Because patient care is only available at these clinics, all cases of clinical malaria could be expected to be captured. Attack rates were determined using population denominators (total camp population, age structure and population of each locality) obtained from the UNHCR registration database and were estimated by taking the number of clinical malaria cases diagnosed at the clinics and dividing by the population within a particular age category over the three day period.

### Ethical Consideration

This survey was a response to a public health emergency and was considered exempt from IRB review by the Centres of Disease Control and Prevention (CDC). Clearance for this survey was received from the Kenya national malaria control program the Division of Malaria Control (DOMC), Ministry of Health, Kenya.

## Results

### Malaria prevalence and attack rate

A total of 324 patients presenting with fever ≥ 37.3°C were tested for malaria infection. Of these, 303 patients were from the four camp clinics and 21 were seen at the camp hospital. Refugees comprised 276 (85%) whilst the remaining 48 (15%) were Kenyan nationals. The majority of the refugees surveyed (50.4%) were Sudanese (Table 1). Among patients of all nationalities, 21% were less than 2 years old, 18% were 2-4 years old, 36% were 5-17 years old and 24% were 18 years old or older. Of the 316 patients for whom gender information was available, 149 (47%) were female. Of the 324 febrile patients tested by RDT, 163 (50%) were positive for *P. falciparum *malaria parasites whilst of the 320 tested by microscopy, 143 (44%) were positive for *P. falciparum*; one patient had a mixed *P. falciparum *and *Plasmodium vivax *infection and one patient was positive for *Plasmodium malariae*. Because 99% (144/146) of infections were *P. falciparum*, the rest of the analysis is done on this species only. Comparing the RDT and microscopy results based on the 320 patients for which both RDT and microscopy were done, the RDTs had a false-negative rate of 4.9% (8 of 162) and a false-positive rate of 14.60% (23 of 158). The RDTs were 94.4% sensitive, comparing well with the performance of ParaCheck,^® ^RDTs when tests were performed by study staff in Kenya [18].

**Table 1:** Number of patients with fever and positive by blood smear for *Plasmodium falciparum *infection at Kakuma Refugee Camp, Kenya, August, 2005 by nationality and by age group

	Number	Total positive	% positive (95% CI)
Nationality			
Sudanese	235	105	44.7 (38.2 - 51.3)
Somali	27	6	22.2 (8.6 - 42.3)
Ethiopian	7	3	42.9 (9.9 - 81.6)
Other	6	3	50.0 (11.8 - 88.2)
Kenyan	47	27	57.5 (42.2 - 71.7)
Age (years)			
0-1	67	9	13.4 (6.3- 24.0)
2-4	58	32	55.2 (41.5 - 68.3)
5-17	117	73	62.4 (53.0 - 71.2)
18+	79	30	38.0 (27.3 - 49.6)
Unknown	1	0	0

Among patients presenting to the hospital, 10 (47.6%) were positive for *P. falciparum *infection while among patients presenting to clinics, 134 (44.8%) were positive. There was significant variation in infection rates among the different age categories in the camp (*X^2 ^*= 45.3, df = 3, P < 0.0001) but no difference among the different nationalities (*X^2 ^*= 9.1, df = 4, P = 0.059). *P. falciparum *prevalence was highest in children 5-17 years of age and lowest in those under 2 years of age (Table 1). Two specimens had only gametocytes. For the remaining samples, the median parasite density was 16,027 per microlitre of blood while the geometric mean was 10,723 parasites per microlitre (range 27-161,680).

**Figure 2:**
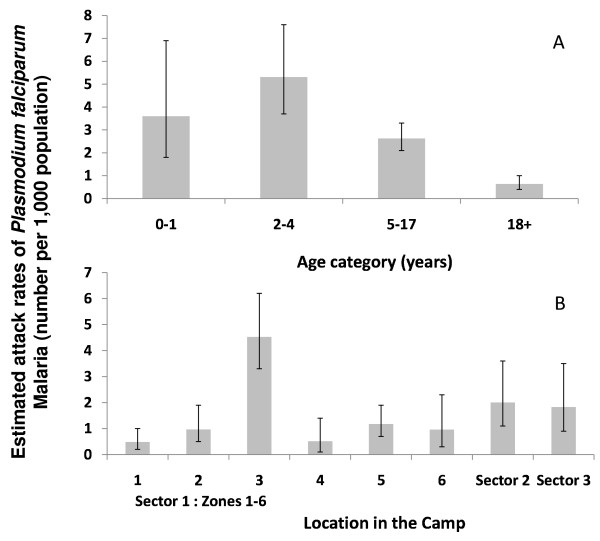
**Attack rates of *Plasmodium falciparum *in the Kakuma refugee camp per 1,000 population over a 3 day period by age group (A) and by location in the camp (B)**.

During days 3 through 5 of the survey, when all camp clinics and the camp hospital were open and data from them aggregated, a total of 128 cases of *P. falciparum *malaria were confirmed among the 89,311 refugees, for an attack rate of 1.4 per 1,000 over the three-day period, or approximately 0.5 new cases per day per 1,000 population in the camp. Attack rate was highest among children aged 2-4 years (Figure 2A). Attack rate varied considerably by reported location of residence within the camp (*X^2 ^*= 23.3, df = 7, P < 0.01), with the highest rates observed in Sector 1 Zone 3, Sector 2 and Sector 3 (Figure 2B).

### Larval habitat survey

**Figure 3:**
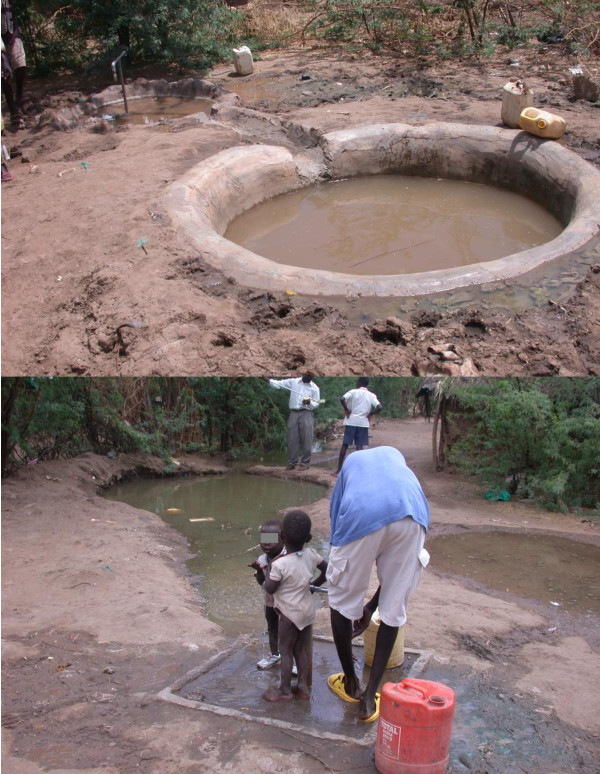
**Examples of tap-stand pits in the Kakuma refugee camp**. Above, cemented pit. Below, soil-lined pit.

**Figure 4:**
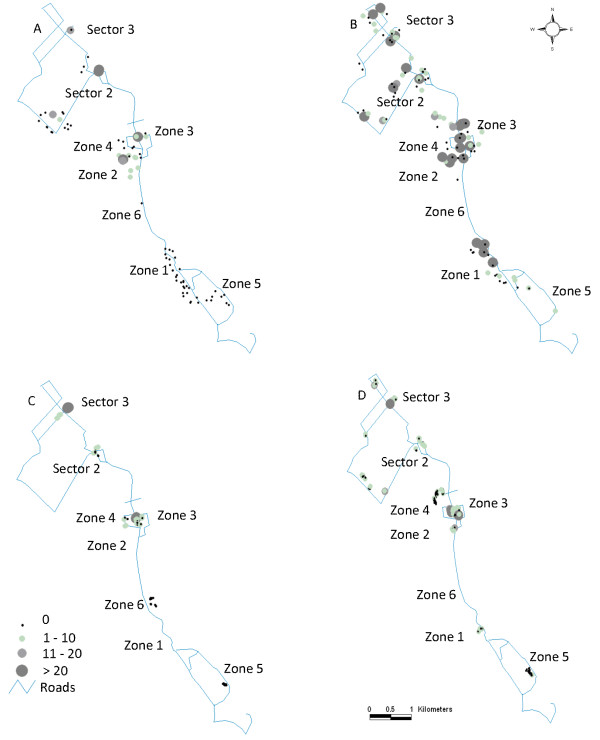
**Spatial distribution of *Anopheles arabiensis *in the Kakuma refugee camp**. Larval density (A. Dry season and B. Wet Season) and indoor adult density (C. Dry season and D. Wet Season) indicated by circles from small circles with no vectors to large circles with more than 20 larvae per dip or 20 adults per house.

During both dry and wet season surveys, we sampled 82 of 255 (32%) tap-stands along with any other potential, ground water habitats for mosquito larvae (Figure 3). Of these, 26/82 (31.7%) tap-stands were not functioning and the pits associated with them were dry. A total of 93 potential larval habitats were encountered and sampled in the dry season, of which 23 (24.7%) were positive for *Anopheles *larvae, yielding 334 larvae and an overall average of 0.4 larvae per dip (Table 2). Morphological identification of the samples indicated that all were of the *Anopheles gambiae *s.l. species complex. Of the 334 larvae collected, 237/328 (72.2%) reacted in PCR and all were *A. arabiensis*. In the wet season, a total of 126 larval habitats were encountered and sampled, of which 56.3% were positive for *Anopheles *larvae, yielding 1,789 larvae and 3.1 larvae per dip on average. Of 883 larvae tested by PCR, 777 (88.0%) reacted and all were *A. arabiensis*. A categorization of habitats by type and summary of sampling results is shown in Table 2. All of the habitats encountered in the dry season were associated directly with tap-stands, and were either cemented pits, soil-lined pits, drainage channels, or run-off puddles whose water source was from the tap-stands. The habitats encountered in the wet season, including habitats that were primarily maintained by water from tap-stands, were mainly cemented pits, soil-lined pits, drainage channels, and run-off puddles (90% of all habitats). Other habitats were those created by rain water: small pools in dry stream beds, wet tire tracks, and roadside puddles (10% of all habitats encountered). The most productive larva habitats were tire tracks and rain fed puddles with densities of the late stages including the 3^rd ^and 4^th ^instars and pupae above 5 per dip, but there were few of these encountered (8.7% of the total habitats observed), consequently making the soil-lined pits (averaging three/dip) the greatest contributor to the local mosquito population when considering their number and density of the late stages (Table 2). The proportion of habitats in the camp that was positive for *Anopheles *larvae was significantly higher in the wet season (56.3%) compared to the dry season (24.7%) (*c^2 ^*= 36.0, df = 1, P < 0.001). Larval density was also significantly higher in the wet season compared to the dry season (Poisson regression, Wald statistic, *c^2 ^*= 57.8, df = 1, P < 0.001). Among the various locations sampled in the dry season survey (Table 3), Sector 2 Phase 1 had the highest larval density followed by Sector 1 Zone 3 (Figure 4A). Four of the locations did not have any positive habitats, namely Sector 1 Zones 1, 4-6. In the wet season, Sector 1 Zone 3 had the highest larval density, followed by Sector 1 Zone 2 (Figure 4B).

**Table 2:** Distribution of positive habitats for *Anopheles *mosquitoes and *Anopheles *larval density* and habitat productivity* in Kakuma refugee camp by season and habitat type

	Dry Season Survey				Wet Season Survey			
Habitat type	No of Habitats sampled	% with larvae	Total larvae (larva density)	Habitat productivity	No of Habitats sampled	% with larvae	Total larvae (larva density)	Habitat productivity
Soil-lined tap-stand pit	46	32.6	216 (0.496)	0.21	44	61.4	764 (3.331)	2.63
Cemented tap-stand pit	30	6.7	9 (0.005)	0.05	34	26.5	167 (0.970)	0.76
Drainage channel	10	30.0	70 (0.414)	0.30	23	73.9	239 (2.000)	1.26
Run off	7	42.9	39 (1.280)	0.20	13	46.2	205 (4.136)	3.17
Roadside puddles	-	-	-	-	5	100.0	115 (6.027)	5.43
Stream bed	-	-	-	-	1	100.0	7 (0.700)	0.6
Tire track	-	-	-	-	6	100.0	292 (12.383)	7.86
TOTAL	93	24.7	334 (0.402)	0.02	126	56.3	1,789 (3.051)	2.23

* Larval density is mean number of all larval stages/dip; while habitat productivity is mean number of the late immature stages (includes from 3^rd ^instar to pupae/dip

**Table 3:** Distribution of positive habitats for *Anopheles *larvae and *Anopheles *larval density (mean larvae/dip) in Kakuma Refugee Camp by season and study site

		Dry Season Survey			Wet Season Survey	
Study Site	No of Habitats sampled	% with larvae	Total larvae (larval density)	No of Habitats sampled	% with larvae	Total larvae (larval density)
Sector 1 Zone 1	7	0.0	0 (0.000)	21	47.6	289 (2.937)
Sector 1 Zone 2	19	47.4	74 (0.426)	17	47.1	297 (3.579)
Sector 1 Zone 3	5	60.0	96 (1.920)	21	71.4	507 (5.397)
Sector 1 Zone 4	1	0.0	0 (0.000)	8	75.0	93 (3.185)
Sector 1 Zone 5	7	0.0	0 (0.000)	5	60.0	17 (1.900)
Sector 1 Zone 6	5	0.0	0 (0.000)	n/a	n/a	n/a
Sector 2 Phase 1	2	100.0	95 (5.432)	15	53.3	92 (1.228)
Sector 2 Phase 2	15	6.7	15 (0.143)	13	46.2	143 (2.460)
Sector 2 Phase 3	6	16.7	1 (0.013)	4	75.0	82 (2.104)
Sector 3	18	27.8	48 (0.316)	20	60.0	269 (2.588)
Sector 4	8	25.0	5 (0.058)	2	0.0	0 (0.000)

### Adult mosquito survey

Of 142 houses sampled in the dry season, a total of 270 *A. gambiae *s.l. (86 males, 184 females) and 1,133 *Culex quinquefasciatus *mosquitoes (429 males, 713 females) were collected (Table 4). Of 264 adult male and female *A*. *gambiae *s.l. tested by PCR, 244 (84.8%) reacted and all were *A. arabiensis*. The mean was 1.9 *Anopheles arabiensis *per house, and the variance was 28.1. Of 177 female *A. arabiensis *tested by sporozoite ELISA, none was positive. Of 301 houses sampled in the wet season, we collected a total of 671 *A. gambiae *s.l. (152 males, 519 females) and 4,059 *Cx. quinquefasciatus *(1,972 males, 2,087 females) (Table 4). Of 715 adult male and female *A. gambiae *s.l. tested by PCR in the wet season, 532 (74.4%) reacted and all were identified as *A. arabiensis*. The mean was 2.2 *A. arabiensis *per house, and the variance was 37.1. Of 502 female *A. arabiensis *tested by sporozoite ELISA, 15 (3.0%) were positive for *P. falciparum *infection. The proportion of houses with *Anopheles *mosquitoes was 36% and 42% in the dry and wet seasons, respectively. Adult *Cx. quinquefasciatus *male and female mosquitoes were present in 78% of houses in the dry season and 82% of houses in the wet season. There was no statistically significant difference between seasons in proportion of houses positive for *A. arabiensis *(*X^2 ^*= 1.4, df = 1, P = 0.23), or indoor density of *A. arabiensis *(Poisson regression, Wald statistic, *X^2 ^*= 0.32, P = 0.57). There was no statistically significant difference between seasons in proportion of positive houses for *Cx*. *quinquefasciatus *mosquitoes between the seasons (*X^2 ^*= 0.0409, df = 1, P = 0.8397) and in number of adult *Cx*. *quinquefasciatus *mosquitoes per house (Poisson regression, Wald statistic, *X^2 ^*= 2.6, P = 0.1043). Among the various locations sampled in the dry season survey, Sector 3 had the highest adult density followed by Sector 1 Zone 3 (Figure 4C). In the wet season, Sector 1 Zone 3 had the highest adult density, followed by Sector 3 (Figure 4D). There was a significant relationship between the number of larval habitats in the site and the number of adult *A. arabiensis *inside houses during the wet season with a 14% increase in adult mosquito density for each additional larval habitat within a site (the 95% Confidence Interval is 6% to 22%, p-value = 0.0001). There was no significant relationship in the dry season.

**Table 4:** Proportion of houses with adult *Anopheles arabiensis *mosquitoes and indoor resting density (no/house) of mosquitoes in Kakuma Refugee Camp by season and location

	Dry Season Survey			Wet Season Survey		
Study Site	No of houses sampled (% positive for *Anopheles)*	Total *Anopheles* (density)	Total *Culex* density)	No of houses sampled (% positive for *Anopheles*)	Total *Anopheles* (density)	Total *Culex* (density)
Sector 1 Zone 1	n/a	n/a	n/a	24 (41.7)	14 (0.58)	218 (9.08)
Sector 1 Zone 2	48 (41.7)	36 (0.75)	503 (10.48)	45 (60.0)	274 (6.09)	814 (18.09)
Sector 1 Zone 3	24 (62.5)	104 (4.33)	247 (9.92)	52 (65.4)	191 (3.67)	447 (8.60)
Sector 1 Zone 4	n/a	n/a	n/a	46 (13.0)	14 (0.30)	186 (4.04)
Sector 1 Zone 5	17 (0.0)	0 (0.00)	207 (12.18)	40 (10.0)	4 (0.10)	202 (5.05)
Sector 1 Zone 6	14 (0.0)	0 (0.00)	68 (4.86)	n/a	n/a	n/a
Sector 2 Phase 1	11 (36.4)	18 (1.64)	68 (6.18)	31 (38.7)	28 (0.90)	498 (16.06)
Sector 2 Phase 2	n/a	n/a	n/a	11 (36.4)	23 (2.09)	1102 (100.18)
Sector 2 Phase 3	n/a	n/a	n/a	17 (29.4)	6 (0.35)	20 (1.18)
Sector 3	17 (70.6)	112 (6.59)	47 (2.76)	31 (71.0)	114 (3.68)	542 (17.48)
Sector 4	11 (0.0)	0 (0.00)	2 (0.18)	4 (50.0)	3 (0.75)	30 (7.50)
TOTAL	142 (35.9)	270 (1.90)	1133 (7.98)	301 (41.9)	671 (2.23)	4059 (13.49)

### Rainfall

**Figure 5:**
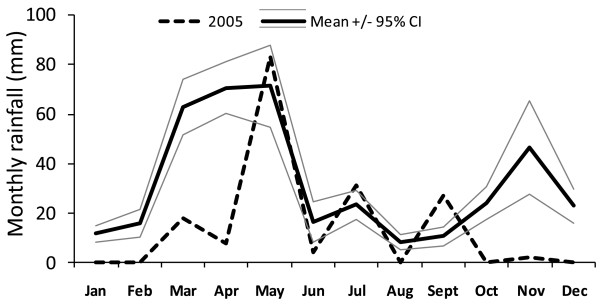
**Average monthly rainfall (2000 - 2007) with 95% confidence interval, Lodwar, Kenya, meteorological station**. Monthly rainfall for 2005 is shown as a dashed line.

Monthly rainfall data from the Lodwar meteorological station revealed a bimodal peak of rain with a longer rain period from March to May and another period in November and December (Figure 5). Rainfall in 2005 (173 mm) was lower than average (216 +/- 119 mm) but within a single standard deviation of the mean. However, rainfall in May, 2005, was 83 mm and the second most for that month in the eight year period from 2000 to 2007.

## Discussion

The Kakuma Refugee Camp is located in an arid environment that, without an exogenous source of water for mosquito larva breeding sites, is unlikely to sustain even intermittent malaria transmission. However, this study describes persistent, malaria attack rates among the refugee population living in the camp and among host nationals living near the camp, with prevalence suggestive of a hyperendemic malaria epidemiology. A subsequent entomological survey, conducted in the dry season, revealed adult anopheline mosquitoes resting inside homes in the camp, when none would be expected given the climatic conditions. Also surprisingly, indoor-resting adult anopheline mosquito densities were nearly as high during the dry season as they were during the wet season. Larval surveys revealed that the vast majority of larvae were found in man-made, tap-stand pits and their drainage systems during both the rainy and dry seasons, indicating that these pits supported mosquito production and, therefore, malaria transmission. They were most likely responsible for most of the malaria-related morbidity and mortality in the rainy season and all malaria in the dry season. Unfortunately, these pits served very little purpose other than as mosquito breeding sites, since the kitchen gardens they were meant to support were rarely implemented by the refugee population (only three such gardens were observed during these surveys).

Human malaria in the refugee camp was entirely due to *P. falciparum *infection albeit one slide was read as mixed infection with *P. vivax *and one was positive for *P. malariae*. *P. vivax *is typically not found in Kenya, but is a common infection in Somalia and Ethiopia, including the arid south-west region of Ethiopia bordering Kenya [5]. Overall results suggest a low level of malaria infection in the camp at the time of sampling (late August 2005), with an estimated attack rate of 0.5 cases per day per 1,000 of camp population. Attack rates varied by age of presenting patient, and were relatively low in the youngest age category (children under two years of age), highest in children and adolescents from two to 17 years old and lowest in the adult age group (age 18 years and older). Young children and pregnant mothers in the camps were provided ITNs, which likely protected these groups from infection. Older children and adolescents have been noted in other populations to be the group least likely to sleep protected by an ITN [19,20]. There was no difference in infection prevalence between the different nationalities. The timing of the investigation was too late to gauge the true picture of the epidemic, to capture the epidemic curve, or to estimate the number of deaths due to malaria; thus, malaria data reported here likely reflect the post-epidemic transmission pattern more typical of the camp's endemicity, when the rains had subsided and the dry season had already commenced, and larval stages of vectors were supported solely by man-made habitats. In January 2007, the established early warning system in the camp reported another increase in clinical malaria cases in the camp (more than 2,500 cases per week) from a usual average of less than 4000 cases per month which subsided by the end of the month. This increase was linked to heavy rains and some flooding in the camp in November and December 2006. Hospital records indicated that there were 8 deaths (2 children and 6 adults) out of 4,800 cases though patients with malaria presented with a wide range of symptoms other than fever including running nose, cough and rash. These results emphasize endemic transmission in the camp with cyclical peaks and not a prevalence of malaria due merely to importation of cases as refugees arrived from other endemic areas.

The rainfall patterns observed from the nearby Lodwar meteorological station confirm a seasonal and modest annual precipitation, but do not indicate that the malaria epidemic in Kakuma refugee camp was due to excessive rainfall in 2005. Rainfall was more constrained seasonally and possibly more intense in that year, however, it did not fall outside of the 95% confidence interval for an eight-year average (Figure 5).

Entomological studies showed that transmission of *P. falciparum *in Kakuma refugee camp was entirely due to *A. arabiensis *as it was the only species of malaria vector found in the area. The lack of livestock animals in the camp likely directed most blood feeding of a relatively zoophilic vector to humans and facilitated parasite transmission. Bovine blood meal sources for vectors were unavailable in the refugee camp because residents were not allowed to keep animals in the camps due to lack of space and the risk of raids by cattle rustlers. The rate of malaria parasite infection in the vectors of 3% in the wet season is consistent with rates quantified elsewhere in *A. arabiensis *populations during the rainy months of the dry regions of northern, sub-Saharan Africa [21]. Indoor-resting densities were modest but probably reflected true population densities as outdoor resting habitat was minimal, due to sparse vegetation and high outdoor temperatures. Even male *A. arabiensis *were caught indoors, suggesting that the indoor environment was a favourable resting habitat for these mosquitoes; otherwise, adults of both sexes of this species commonly rest outdoors [22].

The vector populations were maintained in the camp by a constructed water delivery and catchment system, consisting of a series of tap-stands connected by piping to bore holes, with cemented and soil-lined pits provided to catch spill-over water. These pits had the well-intentioned function of providing irrigation water for kitchen gardens, but such gardens were few in evidence. The most abundant larval habitat was those small bodies of water associated with tap-stand pits, suggesting that the process of pit construction and maintenance underlies the man-made nature of malaria transmission in the camp, and underscores a fundamental conflict between water use and transmission of human malaria. The fact that higher larval densities were found in the much fewer rain-fed puddles and tire tracks is likely a consequence of the rapid concentration of these small environments due to evaporation of water from them, and not to any inherent property of those habitats making them better breeding sites. The similar indoor densities of *A. arabiensis *males and females from the dry (February) and wet (June) samples supports this conclusion; otherwise, rainfall would encourage larger vector populations in June than was actually observed. All available types of larval habitats within the camp were colonized by *Anopheles *vectors and all parts of the camps had at least one house with adult *Anopheles *mosquitoes. However there was aggregation of productive larval habitats in the dry season and houses with highest vector densities in both dry and wet season. This conclusion is supported by the variance to mean ratios of the sampling data [23], which were all much greater than 1, indicating extreme aggregation (i.e., non-random distribution) of larval stages amongst sampled habitats and adults amongst sampled houses. Camp zones and residential sectors with the highest vector densities also had the highest malaria parasite attack rate, suggesting co-aggregation of human exposure to infectious bites and spatial distribution of infected humans. Further analysis of larger data set with adequate spatial information on patients and vectors and habitats within the camp and the surrounding environment where such constructed water supplies systems do not exist would be required to fully understand these relationships.

The number of malaria vectors inside houses at the different sites was positively correlated with the number of habitats in the site. Due to the nature of the sources of these vectors, larval control either by larvicide application or by source reduction or both could be a useful and easily implemented tool for control of malaria in the camp. It could be targeted and monitored readily because of the distinct nature of the habitats. For instance, all pits that were not used for their intended purposes could be filled with soil or modified with a drain outlet to prevent water from accumulating in them. If this recommendation were not feasible, tap-stand monitors should be posted at every tap to manage the immediate environment of the tap-stand by draining pits twice weekly to interrupt the development of larvae, removing any drainage channels emanating from the tap-stand pits, and reporting the presence of any mosquito larvae to the camp authority for targeted larviciding.

Harvesting waste water from tap-stands makes practical sense given common water shortages in the area. If in use and emptied regularly, these tap-stand pits likely would not have had sufficiently stable water sources for the mosquitoes to complete their immature life stages. Because these tap-stand pits were numerous and remained filled with water at all times, there was a year round presence of vector breeding sites throughout the camp leading to a year round production of malaria vectors and the resultant phenomenon of malaria endemicity in an area with otherwise no malaria or at worst seasonal malaria. These findings underline the relevance of monitoring environmental impact of interventions; an old lesson worth heeding.

## Competing interests

The authors declare that they have no competing interests.

## Consent

Blood smears were ordered during clinic visits for patient care purposes and were taken on verbal agreement with the patient. Non-identifying surveillance information from those blood smears are included in this report. Consent was therefore not required.

## Authors' contributions

MNB participated in the design and implementation of the entomology surveys, laboratory analysis of mosquito samples, data analysis and presentation and drafted the manuscript. WA Secured clearances for the study and participated in study design and implementation, and data analysis. MO was involved in the implementation of the entomology surveys and data analysis including the development of maps and figures. DS was involved in the implementation of the entomology surveys. SCE was involved in the implementation of the surveys.

DK contributed to the design of both the malaria and entomology surveys and participated in the malaria data collection. EDW participated in study design, laboratory analysis of mosquito samples, data analysis and manuscript writing. HW participated in the design and implementation of the surveys. HB was responsible for the co-ordination of the study and also contributed to the study design. GA was responsible for the design of the malaria survey and contributed to data analysis and presentation. MC participated in the collection and analysis of the malaria data. MW participated in the collection and analysis of the malaria data. RB participated in study co-ordination, data analysis and manuscript development. MJH participated in the design of the surveys, and contributed to data analysis and presentation and manuscript development. All authors read and approved the final manuscript.
